# Underwater endoscopic mucosal resection of an incompletely resected superficial non-ampullary duodenal epithelial neoplasm using the loop-and-let-go technique: A case report

**DOI:** 10.1097/MD.0000000000024041

**Published:** 2021-01-15

**Authors:** Dong Hyun Kim, Seon-Young Park, Eunae Cho, Chang Hwan Park, Hyun Soo Kim, Sung Kyu Choi, Jong Sun Rew

**Affiliations:** Division of Gastroenterology, Department of Internal Medicine, Chonnam National University Hospital and Medical School, Gwangju, South Korea.

**Keywords:** duodenal neoplasm, duodenoscopy, endoscopic mucosal resection, residual neoplasm

## Abstract

**Rationale:**

Endoscopic resection of superficial non-ampullary duodenal epithelial neoplasm (SNADEN) is a challenging procedure owing to the high recurrence rate and considerable incidence rate of adverse events.

**Patient concerns:**

SNADEN accidentally found during a medical examination in a 56-year-old man

**Diagnosis:**

The patient was diagnosed as having a 20-mm-sized flat elevated SNADEN at the superior duodenal angle.

**Interventions:**

First, we tried to perform conventional EMR (CEMR). However, the submucosal injection interrupted the endoscopic view and did not provide enough space for CEMR because of its angulated location. Therefore, we chose to perform endoscopic resection using the “loop-and-let-go” technique. Follow-up duodenoscopy after 2 days revealed post CEMR ulcer with suspicious remnant lesion. Underwater endoscopic mucosal resection (UEMR) was successfully performed 3 months after the first session of endoscopic resection.

**Outcomes:**

Complete endoscopic en bloc resection and histological complete resection were achieved with UEMR. Follow-up duodenoscopy revealed no recurrence.

**Lessons:**

Step-by-step endoscopic treatment with UEMR following loop-and-let-go technique may be a good strategy for SNADEN over 20-mm in diameter.

## Abstract 2

Supplemental Digital Content is available in the text

## 1 Introduction

Recently, the incidence of superficial non-ampullary duodenal epithelial neoplasm (SNADEN) has increased because of the popularization of gastrointestinal endoscopic examination with the increasing need for diagnostic or screening purposes. Even if no definite treatment guidelines have been established regarding the indication for endoscopic therapy and selection of the treatment strategy, less invasive treatment modalities such as endoscopic mucosal resection (EMR) and endoscopic submucosal dissection (ESD) are preferred and thus frequently performed by endoscopists. EMR is the most common technique; however, the recurrence rate is high (5%–37%), and the procedure is accompanied by a considerable rate of adverse events such as bleeding and perforation.^[1–5]^ ESD is also a frequently used technique and could improve the rate of complete resection. However, this procedure needs a considerably prolonged procedure time and is associated with a higher incidence of adverse events such as perforation and bleeding.^[3,6]^ In particular, the superior duodenal angle (SDA), an area with a narrow lumen and acute angle, makes complete resection difficult to perform. Recently, underwater EMR (UEMR) has been used in the treatment of challenging lesions in the duodenum and colon, including the appendiceal orifice and rectum reaching the dentate line. The treatment results seem good, providing high complete resection rate and low adverse event rate.^[7–10]^ Recently, Iwagami et al^[11]^ reported the effectiveness and safety of UEMR in SNADEN when other efficacious procedure are unavailable.

The loop-and-let-go technique is rarely used to remove large colonic lipoma or subepithelial lesions of the gastrointestinal tract.^[12,13]^ To our knowledge, the loop-and-let-go technique has not been used in SNADEN, but it is known as a method of safe tumor removal, reducing the risk of bleeding or perforation.^[12,13]^ Here, we report a case of complete resection of a SNADEN using UEMR after incomplete resection with loop-and-let-go technique.

## 2 Case report

**Figure 1:**
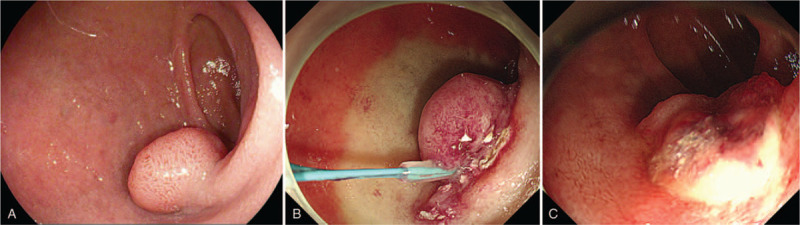
Initial duodenoscopy image showing a 20- × 15-mm-sized flat round elevated SNADEN (A). Endoscopic resection of the SNADEN using the loop-and-let-go technique (B). Post-procedural duodenal ulcer with remnant lesion 2 days after the endoscopic resection (C).

**Figure 2:**
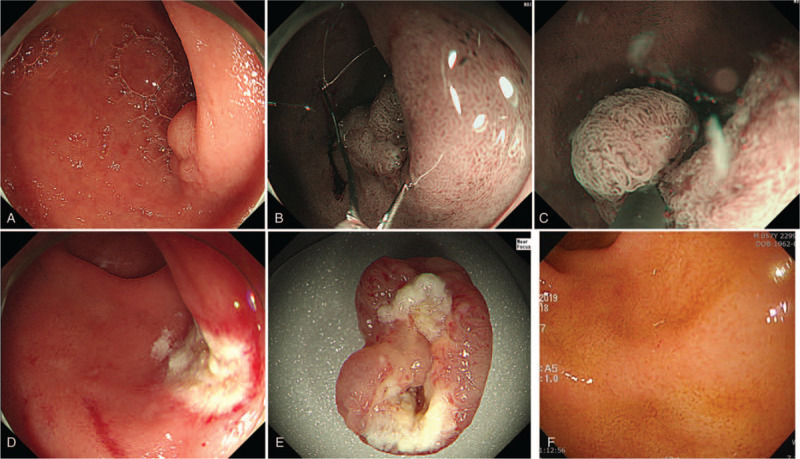
Duodenoscopy image 3 months later, showing a flat elevated remnant SNADEN approximately 15 × 12 mm in size (A). Snaring of the remnant SNADEN (B). Captured SNADEN by an endoscopic snare underwater (C). Completely resected SNADEN after UEMR in the endoscopic view (D). Gross image of the completely removed SNADEN (E). Follow-up duodenoscopy image showing no recurrence of the SNADEN (F).

A 56-year-old man underwent screening esophagoduodenoscopy, which showed a 20- × 15-mm-sized flat round elevated mucosal lesion in the SDA. The histologic finding based on the forceps biopsy suggested a tubulovillous adenoma with high-grade dysplasia. First, we tried to perform conventional EMR (CEMR). However, the submucosal injection interrupted the endoscopic view and did not provide enough space for CEMR because of its angulated location. Therefore, for the lesion, we instead chose to perform endoscopic resection using the loop-and-let-go technique (MAJ-254; Olympus, Tokyo, Japan). Follow-up duodenoscopy after 2 days revealed ulceration caused by the endoscopic resection and a remnant SNADEN (Fig. 1). We decided to perform a second session of endoscopic resection 3 months after the first procedure. The endoscopic view revealed a flat elevated lesion approximately 15 × 12 mm in size with an artificial ulcer scar in the SDA. We performed UEMR without submucosal injection instead of CEMR because the SNADEN was acutely angulated. Cap-assisted duodenoscopy (GIF HQ290, Olympus) with narrow-band imaging was used to confine the margin of the SNADEN. UEMR was performed using a water jet pump (Olympus device), crescent-type snare (Olympus device), and VAIO 300D (ERBE Co. Ltd., Tubingen, Germany) with a high-frequency generator. The settings of the VAIO 300D were as follows: Endocut-Q, effect 2, incision time 3, and incision interval 5. It took 90 s after initial water infusion to complete removal of the lesion (Supplementary video {Video of the UEMR without submucosal injection for resection of the remnant SNADEN, which was not completely resected using endoscopic resection with the loop-and-let-go technique.}, http://links.lww.com/MD/F515). No adverse events occurred, including bleeding or perforation. We did not use any additional device for the prevention of adverse event. After 2 months, follow-up endoscopic and histological examinations revealed no recurrence in the site of the resected lesion (Fig. 2).

## 3 Discussion

This case demonstrates the effectiveness of UEMR for the treatment of SNADEN located at SDA. CEMR for duodenal lesions is often difficult because the duodenal lumen is acutely angulated and narrow, and the submucosa in the duodenum is fibrotic and highly vascular, resulting in poor lifting by submucosal injection. Moreover, inadequate injection sometimes makes snaring difficult because of luminal narrowing.^[1–5]^ ESD is performed for complete resection of duodenal neoplasms. However, this procedure needs a considerably prolonged procedure time with relatively higher incidence of adverse events such as perforation and bleeding.^[3,6,14]^ Pocket creation method is another option for resection of duodenal neoplasm to prevent adverse events such as perforation, even though it showed relative high perforation rate and prolonged procedure time.^[14]^

We tried to perform CEMR for SNADEN. However, we changed the method of endoscopic resection with loop-and-let-go technique, as the submucosal injection interrupted the endoscopic view and did not provide enough space for CEMR. Endoscopic resection using the loop-and-let-go technique is rarely used to remove large polyps or subepithelial lesions, which may avoid possible thermal injury that leads to bleeding or perforation.^[15,16]^ As we could not achieve complete resection using loop-and-let-go technique, we performed UEMR as 2nd session of endoscopic procedure. Nowadays, UEMR is selectively performed for the management of tumor of stomach, colon, and duodenum, especially in cases of difficult to preform CEMR or ESD due to location or submucosal fibrosis.^[7–11,17]^ Binmoeller et al developed UEMR as a treatment for SNADENs,^[18]^ which showed a high complete resection rate and low adverse events rates for SNADENs over 20 mm in size. And Yamasaki et al also showed a good treatment outcomes for SNADENs under 20 mm in size.^[7]^ Recently, Iwagami et al demonstrated good efficacy of UEMR for 162 patients with SNADEN with short procedure time and no perforation.^[11]^ Previous reports for UEMR showed a good treatment outcomes with relatively short procedure time and low adverse events rate.^[7,11,18]^ However, it depends on the tumor size of SNADENs; En bloc resection rate for ≥20 mm in size was 14%, while those for < 20 mm in size was 79% to 87%.^[7,11]^ Median procedure time is 65 minutes for SNADENs ≥ 20 mm in size, while it decreases by 5.9 minutes for SNADENs < 20 mm in size.^[7,18]^ UEMR has benefits for removal of SNADEN as follows;

1. superficial lesions float up into the snare as protruding lesions in underwater conditions,
2. the duodenal angles become obtuse,
3. the narrow duodenal lumen is constantly distended.
4. The underwater resection decreases the thermal damage to the duodenal wall, which helps to prevent delayed perforation, and
5. the resection plane by UEMR is superficial, in which submucosal vessels usually remained within the resection wound, whereas the submucosal vessels were disrupted in CEMR.^[7]^

However, there are reports of delayed bleeding, water intoxication syndrome, and aspiration pneumonia, which requires attention.^[7,11,18]^ As UEMR has been performed recently, long-term follow-up outcomes are required to validate the efficacy and safety for UEMR.

We performed step-by-step endoscopic treatment with UEMR following loop-and-let-go technique for SNADEN over 20-mm in diameter. Even though we could not achieve complete resection using Loop-and-let-go technique, the size of SNADEN was reduced, which might have contributed to the success of the second session procedure with UEMR.

## 4 Conclusion

Step-by-step endoscopic treatment with UEMR following loop-and-let-go technique may be a good strategy for SNADEN over 20-mm in diameter.

## Author contributions

DHK performed the UEMR. EC performed endoscopic resection using the loop-and-let-go technique, CHP, HSK, SKC, and JSR collected the patient's data and contributed to the writing of the manuscript. DHK and SYP designed the research study and wrote the manuscript.

## Abbreviations

Abbreviations: CEMR = conventional endoscopic mucosal resection, EMR = endoscopic mucosal resection, ESD = endoscopic submucosal dissection, SDA = superior duodenal angle, SNADEN = superficial non-ampullary duodenal epithelial neoplasm, UEMR = underwater endoscopic mucosal resection.
